# Bias due to MEasurement Reactions In Trials to improve health (MERIT): protocol for research to develop MRC guidance

**DOI:** 10.1186/s13063-018-3017-5

**Published:** 2018-11-26

**Authors:** Lisa M. Miles, Diana Elbourne, Andrew Farmer, Martin Gulliford, Louise Locock, Jim McCambridge, Stephen Sutton, David P. French

**Affiliations:** 1 Manchester Centre for Health Psychology, University of Manchester, Oxford Road, Manchester, UK; 2 Department of Medical Statistics, London School of Hygiene and Tropical Medicine, Keppel Street, London, UK; 3 Nuffield Department of Primary Care Health Sciences, University of Oxford, Oxford, UK; 4 School of Population Health and Environmental Sciences, King’s College London, London, UK; 5 Health Services Research Unit, University of Aberdeen, Aberdeen, UK; 6 Department of Health Sciences, University of York, York, UK; 7 Department of Public Health and Primary Care, University of Cambridge, Cambridge, UK

**Keywords:** Measurement, Reactivity, Measurement reactions, Guidance, Trials, Bias, Hawthorne effect

## Abstract

**Background:**

There is now clear systematic review evidence that measurement can affect the people being measured; much of this evidence focusses on how asking people to complete a questionnaire can result in changes in behaviour. Changes in measured behaviour and other outcomes due to this reactivity may introduce bias in otherwise well-conducted randomised controlled trials (RCTs), yielding incorrect estimates of intervention effects. Despite this, measurement reactivity is not currently adequately considered in risk of bias frameworks. The present research aims to produce a set of guidance statements on how best to avoid or minimise bias due to measurement reactivity in studies of interventions to improve health, with a particular focus on bias in RCTs.

**Methods:**

The MERIT study consists of a series of systematic and rapid reviews, a Delphi study and an expert workshop to develop guidance on how to minimise bias in trials due to measurement reactivity. An existing systematic review on question-behaviour effects on health-related behaviours will be updated and three new rapid reviews will be conducted to identify (1) existing guidance on measurement reactivity; (2) systematic reviews of studies that have quantified the effects of measurement on outcomes relating to behaviour and affective outcomes in health and non-health contexts and (3) trials that have investigated the effects of objective measurements of behaviour on concurrent or subsequent behaviour itself. A Delphi procedure will be used to combine the views of experts with a view to reaching agreement on the scope of the guidance statements. Finally, a workshop will be held in autumn 2018, with the aim of producing a set of guidance statements that will form the central part of new MRC guidance on how best to avoid bias due to measurement reactivity in studies of interventions to improve health.

**Discussion:**

Our ambition is to produce MRC guidance on measurement reactions in trials which will be used by future trial researchers, leading to the development of trials that are less likely to be at risk of bias.

**Electronic supplementary material:**

The online version of this article (10.1186/s13063-018-3017-5) contains supplementary material, which is available to authorized users.

## Background

Measurement reactivity has been defined as being present where measurement in a research project results in changes in the people being measured [1]. The changes can be behavioural, emotional or cognitive (e.g. beliefs). Concepts akin to measurement reactivity have been recognised for many years. For instance, it was shown over 40 years ago that being interviewed on intention to vote in elections alters the likelihood of actually doing so [2]. Measurement reactivity has been studied across many disciplines where several terms have been used to describe this phenomenon, including ‘assessment reactivity’, ‘mere-measurement’, ‘question-behaviour effect’ and ‘self-generated validity’ [1].

There is now clear evidence from systematic reviews that measurement can affect behaviour [3–7]. Much of this evidence derives from studies where people who were asked to complete a questionnaire showed changes in behaviour relative to a control group. Questions answered for research assessment purposes may stimulate new thinking about a behaviour. These questions may then be a prelude to action. Overall, the main findings of these systematic reviews are remarkably consistent, and can be summarised: (1) there are overall effects of asking questions on objective and subjective measures of behaviour but these effects are typically small; (2) there is considerable heterogeneity in effects on behaviour across studies in the reviews; (3) few of the primary studies in the reviews have low risk of bias, with a lack of pre-registration of protocols as a particular weakness and (4) publication bias is present in the reviews, but not of sufficient extent to reduce best estimates of effects on behaviour to zero.

Further examples of experimental studies have provided evidence that measurement can affect research participants. There is a large body of literature showing that repeated completion of quality of life questionnaires can produce a ’response shift’ in a person’s frame of reference when judging their quality of life [8]. Presenting questions in different orders in questionnaires affects responses to those questionnaires [9]. For example, it appears that when people complete anxiety questionnaires on multiple occasions, they score higher on the first occasion of measurement [10]. By contrast, when anxiety measures are placed at the end of questionnaires this results in higher anxiety scores than when they are placed at the beginning of questionnaires [11]. In addition to the effects of answering questions, there is also some evidence that objective research assessments, such as electronic monitoring of behaviour may produce similar reactions [12].

These ideas are related to the broader ’Hawthorne effect’ [13] which is used to refer to the impact of observation and other forms of monitoring on participants in research. The Hawthorne effect appeared in a research publication 65 years ago [13] and is in widespread use. Despite the common use of this term, there is little dedicated research into its extent and nature, and it has been proposed that more precise terms are needed to develop understanding of research participation effects [14].

Qualitative studies of completion of questionnaires [15] and experiences of participation in randomised controlled trials (RCTs) [16] have shed light on how measurement can produce changes in people. For instance, they have shown how the act of completing a questionnaire may create new beliefs [15]. They have also shown how participants’ understanding of questionnaires as research tools affects how people complete questionnaires, and how they subsequently behave and feel. There is also evidence that people taking part in research do so partly because they see personal benefit in doing so, including access to monitoring of their own health [16].

The challenges associated with measurement reactivity are pertinent for RCTs, especially in the context of behaviour change, public health and health service research. Changes in measured behaviour and other outcomes due to measurement reactivity may introduce systematic error or bias, making it difficult to distinguish true change in outcomes arising from the intervention, from change due to a combination of intervention and measurement. If there are similar levels of reactivity between experimental groups in a RCT it might be considered that the true effects of interventions are safeguarded by randomisation, but this does not take into account the possibility that measurements might interact with interventions to either strengthen or weaken the observed effects, and, therefore, lead to biased estimates of effect [17, 18]. For example, research measurement could prepare participants to be more receptive to an intervention by prompting contemplation which serves as a preparation for behaviour change [17, 18].

Similarities between the contents of research measurements and interventions also provide *prima facie* grounds for concern over risk of bias. For example, there is systematic review evidence that pedometers, particularly where the measurements are not concealed, may be effective intervention tools by promoting self-monitoring of behaviour [19]. Given this, it becomes problematic to use pedometers as baseline and outcome measures in studies of interventions which aim to increase physical activity via participant self-monitoring. In this situation, estimates of effectiveness are likely to be biased towards the null, as both intervention and control groups are exposed to the pedometer acting as a self-monitoring intervention. This implies contamination of intervention content if the pedometer itself is an intervention component, and the control group participants are exposed to it. Where it is not an intervention component, the intended experimental contrast may be thwarted, and any effects of physical activity interventions should instead be interpreted to mean how much they perform better than pedometer and other control group content.

Concerns around bias are also warranted where measurement is unbalanced across randomised groups, with one group being measured more than another. For example, there is often integration of measurement and intervention in eHealth intervention trials. In such studies, participants in only one experimental condition may be asked to (1) complete measures of motivation or behaviour to allow tailoring of interventions or (2) complete ongoing measurements using technology such as an application (app), whilst participants in the control condition are not asked to complete these additional measures. Such trials are increasingly common; a 2010 systematic review of computer-tailored interventions identified 88 eligible trials [20].

Systematic reviews indicate a standardised mean difference (SMD) = 0.09 in behaviour between groups that are asked to complete measures in relation to health-related behaviours relative to groups that do not complete measures [4]. Such effects appear to be inconsistent across settings, populations and measures. Given that systematic reviews of complex behaviour change interventions often report effects of the order of SMD = 0.20 to 0.30 [21–23], there is clearly potential for proportionately large effects of bias of RCT results. The biasing effects of research measurements, where they exist, are likely to be variable across populations, behaviours, interventions and outcomes as well as the particular measurement methods used [17]. They may also operate across study designs and interact with existing forms of bias [24]. Whilst there is increasing scrutiny of the mechanisms through which measurement can affect behaviour [7], there appears to have been little systematic consideration given to identifying the precise circumstances in which measurement reactivity can occur and how it might lead to bias. Importantly, there is also little agreement on how to predict the likelihood or extent of reactivity, or how to control for it in the design of RCTs and other interventional studies.

One potential solution to this problem has been offered in the Solomon four-group study design [25]. In a Solomon design, participants are randomly allocated to one of four arms: (1) experimental group with baseline assessment; (2) experimental group without baseline assessment; (3) control group with baseline assessment or (4) control group without baseline assessment. This design estimates the effects of baseline assessment and can assess interactions between the intervention and baseline assessment [26]. A systematic review [17] of evidence from Solomon four-group studies identified 10 studies but overall there were too few studies of high quality to infer conclusively that biases stemming from baseline research assessments do or do not exist. Overall, Solomon four-group studies have not been widely used in social and health science studies with behavioural outcomes, at least partly due to the difficulty in justifying such a design in the absence of data on the likelihood of measurement reactivity, and hence the particular threats to valid inference. Furthermore, Solomon four-group studies require a substantial increase in sample size and so are costly.

In sum, there is now good evidence that measurement is not an inert procedure (research participants can react to being measured), and also that it has the potential to cause bias in research [1]. Despite this, measurement reactivity has generally been ignored in discussions of how to reduce bias in trials. There is no agreed set of practices for conduct, reporting or analysis of measurements that allow the potential for bias to be appreciated. To the authors’ knowledge, no guidance on handling or minimising the impact of measurement reactivity in RCTs or other research studies has been produced beyond a brief set of considerations for trial design produced by members of this research team [18].

The MEasurement Reactions In Trials (MERIT) study has been designed to produce a set of guidance statements on how best to avoid or minimise bias due to measurement reactivity in studies of interventions to improve health, with a particular focus on bias in RCTs. The focus on trials is justified by the central importance of trials evidence for healthcare decision-making, although we recognise that measurement reactivity is likely to cause bias in research using other study designs. The MERIT study was commissioned in response to a call by the Medical Research Council (MRC)/ National Institute of Health Research (NIHR) Methodology Research Programme which determined that the potential for measurement reactivity to cause bias is a key area of uncertainty. The MERIT study consists of a series of systematic and rapid reviews, an international Delphi procedure, and an expert workshop to develop guidance to the research community. In this paper we describe the protocol for the MERIT study.

### Aim of the study

The aim of the MERIT study is to develop expert guidance on how to avoid bias due to measurement reactivity in RCTs of interventions to improve health. To achieve this aim, the following objectives will be addressed:

1. To identify and summarise key background literature examining measurement reactivity
2. To determine the scope of the guidance that would best meet stakeholder needs through a Delphi procedure
3. To produce guidelines through an expert workshop

## Methods

### Preliminary framework

To help structure ongoing discussions around the research evidence that will underpin the development of guidance, the MERIT team is developing a conceptual framework. Table 1 shows an overview of a preliminary version of the conceptual framework that is a starting point for the study. This framework aims to map out how measurement changes people, the sorts of biases that are likely to arise from this, and the circumstances that make biases more or less likely to occur. To date, the framework has been developed within the MERIT research team; we expect it to facilitate thinking around the development of guidance. The framework will be subject to further rounds of iteration as the project progresses. Further feedback and refinement is expected from the Delphi procedure and the expert consultation workshop, described in further detail below. We anticipate a more elaborated version of this framework to be an output of the MERIT study; this will be published as part of the final MRC guidance.

**Table 1:** Bias in trials due to measurement: a preliminary framework

Our preliminary framework consists of three elements:	
(A) What sorts of bias can arise from measurement reactivity (and what are the relationships to the existing well-known forms of bias)?	
1. Main effects of measurement on trial outcome	
2. Where there is an interaction between measurement and trial-arm status on outcome	
3. Where measurement results in study dropout	
(B) How does measurement produce changes in people (i.e. what are the mechanisms)?	
1. Measurement changes the performance of that behaviour or reports of performance of that behaviour	
2. Measurement changes emotional states or reports of emotional state	
3. Measurement changes questionnaire or study completion rate	
(C) What are the characteristics of measurement, people and context that can lead to or moderate the risk of such biases (i.e. when might measurement reactivity be anticipated)?	
1. Features of measurement that produce reactivity	
2. Features of outcome measurement (may be the same as above)	
3. Features of participants being measured	
4. Other features of context surrounding measurement or trial not captured in other categories	

### Background literature examining measurement reactivity

In addition to the conceptual framework, a number of literature reviews are being conducted to map out what is known and unknown about the nature of measurement reactions. One existing systematic review is being updated and three new rapid systematic reviews [27] are being conducted. The new rapid reviews will be conducted in parallel, using formal database searches and contacts with leading individuals within this field internationally.

#### Systematic review of the question-behaviour effect on health-related behaviours

An existing systematic review [4] of the question-behaviour effect on health-related behaviours will be updated. This systematic review is particularly relevant because it focusses on health contexts and included the most thorough assessment of risk of bias of existing reviews on this topic [28]. There is a need to update this search given that the original search for this review was conducted in December 2012. Importantly, more recently conducted studies have been published with lower risk of bias than earlier studies [29, 30].

The update will use the databases MEDLINE, PsycINFO, Embase and Cochrane Central Register of Controlled Trials (CENTRAL). The same search strategy and methodology will be used for the update as in the previous systematic review, and a forward citation search of the original systematic review [4] will be completed. RCTs including factorial (Solomon) designs will be included; non-randomised or quasi-randomised trials will be excluded. Included measurement conditions will include interviews and questionnaires assessing cognitions and/or behaviours, using pencil and paper or online methods. Measurement conditions that include elements of self-monitoring or participant-feedback (for example, blood pressure monitoring) will be excluded. For inclusion, trials will require a no measurement or alternative measurement control group as comparators. Primary outcomes are all objectively or subjectively measured health-related behaviours, including proxy measures of health behaviour. Predictive measures of behaviour, such as intention and self-efficacy, will be secondary outcomes. Risk of bias will be appraised using the Cochrane Collaboration tool [31]. PRISMA (Preferred Reporting Items for Systematic Reviews and Meta-Analyses) guidance [32] will be used for reporting the systematic review.

#### A rapid review to identify existing systematic reviews of studies that have quantified the effects of measurement on outcomes relating to behaviour and affective outcomes in health and non-health contexts

Reviews that include subjective and objective research measurements and different modalities of measurement (for example, questionnaires, pedometers and physiological testing) will be included. The following databases will be searched, limited to articles published in English in the last 10 years with no limit on document type: PsycINFO; Medline; Cochrane Database of Systematic Reviews and PROSPERO (for ongoing reviews). Titles and abstract will be screened by one reviewer, with a second reviewer independently screening 50% of titles and abstracts. Full-text versions of potentially relevant articles will be obtained and independently screened by two reviewers for relevance, as well as quality based on the AMSTAR (Assessing the Methodological Quality of Systematic Reviews) framework [33]. Results of the reviews will be tabulated to allow comparison of aims, scope, methods, quality and findings. A narrative synthesis of the review findings will be produced with a greater emphasis on reviews of higher quality.

#### A rapid review of trials that have investigated the effects of objective measurements of behaviour on concurrent or subsequent behaviour itself

Much of the existing literature on measurement reactivity focusses on the effects of questionnaire measurement of behaviour on subsequent behaviour. By contrast, this rapid review will address a key gap in knowledge, as objective measures of health behaviour are increasingly being used with development in information technology. For example, accelerometers to measure physical activity, electronic monitoring of medication adherence or taking photographs of food to measure food intake all provide alternatives to reliance on retrospective self-report. We will improve on existing reviews in this area by examining the extent to which participants are blinded to outcome assessment moderates the effects of measurement on behaviour. Quantifying the effects of objective measures may facilitate statistical adjustments to take place in studies of the effects of behavioural interventions.

Relevant trials will be identified by searching PsycINFO and Medline for relevant articles published in the last 10 years, in English, with no limit on document type. The search strategy will combine terms for measurement methods and target behaviour and include experimental between-individuals or within-individuals designs. The reference lists of identified papers as well as the rapid review of systematic reviews will be handsearched for additional relevant papers.

Titles and abstracts will be screened by one reviewer, with a second reviewer independently screening 50% of titles and abstracts. Full-text versions of potentially relevant articles will be obtained and independently screened by two reviewers. For the final set of papers, data will be extracted on to a standardised form by one reviewer, and the key information will be checked by a second reviewer. Risk of bias of included studies will be assessed according to the Cochrane Handbook for Systematic Reviews [31]. Results of the studies will be tabulated to allow comparison and findings will be reported according to PRISMA guidelines [32]. If the nature and amount of studies is adequate, meta-analysis will be conducted using a random effects model to compare objective measurement versus non-measurement conditions; possible sources of heterogeneity will be investigated.

#### Rapid review to identify existing guidance on measurement reactivity

To the authors’ knowledge, no formal guidance on handling or minimising the impact of measurement reactivity in research studies has been produced. To investigate this assumption, a rapid review of existing guidance will be completed. A search for existing guidance on measuring reactivity will be conducted using Medline. CONSORT (Consolidated Standards of Reporting Trials) Statements, MRC framework/guidance on complex interventions (all versions) and MRC guidance on process evaluation in trials will also be examined for existing guidance.

### Delphi procedure to determine the scope of the guidance

This study will use the Delphi method [34] to explore and as far as possible combine the views of experts to reach agreement on the precise issues that the guidance will cover, i.e. the scope of the guidance statements. Use of a Delphi procedure can engender group ownership and enable cohesion among participants with diverse views [35] and allows input from experts internationally without geographical constraints. We recognise that the subject matter of this study may be somewhat challenging for participants, so there may be limits to the degree of consensus possible. If agreement is not reached, the Delphi process will nevertheless identify where consensus has not been possible. Participants will complete at least two rounds of a brief online questionnaire, over a period of approximately 12 weeks in Spring/Summer 2018. The objectives of the Delphi procedure are:

1. To seek expert opinion from stakeholders on the specific topics where guidance on measurement reactions is needed and likely to produce the largest benefit.
2. To elicit expert feedback on the preliminary framework of measurement effect.
3. To identify key background literature and expertise on measurement reactivity

Delphi participants will be purposively recruited. Suitable experts will be identified by examining authorship of studies cited in the rapid review of systematic reviews, as well as knowledge within the multidisciplinary research team. Invitations to the first round of the Delphi will also ask for recommendations of colleagues or contacts who might contribute usefully to the project. The aim is to identify individuals with expertise relating to measurement reactivity and trial design, conduct and analysis to gain experience and knowledge relevant to the content of the guidance. We also aim to identify individuals who are likely to be key users of the final guidance, including those involved in research synthesis and funding, so that its content reflects stakeholder needs, as well as those who are likely to disseminate the guidance. We will also seek to identify public/patient representation to allow the experiences of people who take part in research, particularly in trials, to be reflected in the final guidance, though this is expected to be challenging. The list of categories of expertise identified for the Delphi participants is available in Additional file 1.

Given the likely heterogeneity in expertise of the sample, we will attempt to recruit 40 individuals, which is a larger sample size than is typically used [36]. The aim is to minimise participant burden to maximise response rate, and to be as transparent as possible in the processes that will be used to prioritise topics that guidance might consider. Participants will be informed explicitly how the data will be used in the MERIT study and asked to provide informed consent before completing the first questionnaire. Potential participants will not be pursued beyond two reminders. Anonymity and confidentially of all responders and non-responders will be maintained.

The first round of the Delphi procedure will ask participants to indicate the specific topics where guidance is needed and likely to produce the largest benefits. Views will be elicited in this first round using a small number of open-ended questions to gain insight into what sorts of bias can arise from measurement reactivity, the mechanisms by which measurement produces changes in people, and the characteristics of measurement and context that can lead to such biases. This will help to inductively arrive at an overview of where guidance would be most useful. Suggestions will also be sought on key literature on measurement reactivity.

The second round of the Delphi process will summarise the results of the first round, to produce a list of specific topics that guidance might consider, and where it is most needed. Other topics will be included based on suggestions from the applicants where there were omissions in the first round. Participants will be asked to rate their agreement with suggestions for inclusion in guidance, as well as provide open-ended comments if they think any other key issues are missing. If required, a third Delphi round will summarise the results of the second round and participants will be asked to rate agreement.

Delphi participants will be asked to indicate if they are willing to participate in the expert consultation meeting, what issues they would find particularly interesting, and suggest other individuals who could usefully provide input in the subsequent expert consultation meeting. Thus, the Delphi process should identify the specific topics where guidance on measurement reactions is needed and likely to produce the largest benefits on RCT design and conduct.

### Producing guidance from expert consultation

A two-day face-to-face expert consultation meeting will be held in Manchester, UK, in autumn 2018. The central objective of the consultation meeting is to produce a set of guidance statements that have the support of the majority of meeting participants. These statements will form the central part of the MRC guidance.

The topics under discussion at the consultation meeting are likely to include many aspects of the preliminary framework, which will be refined according to participant responses from the Delphi procedure. Topics are likely to include the sorts of biases that can arise from measurement reactions, the circumstances in which they are more likely to arise, the mechanisms by which measurement reactions operate and features of study design and/or analysis that can be used to avoid or minimise risk of such bias in trials.

A number of steps will be followed to prepare for the consultation meeting, with some flexibility where appropriate:

1. Identification of approximately six key topics that require guidance, with members of the research team (or nominees) being identified as leads for groups focussing on each issue
2. Recruitment of five to six people to form groups to focus on each of the six key issues, based partly on preferences indicated by participants in the Delphi procedure, recommendations by Delphi participants and authors of key literature. We will purposively recruit to ensure diversity within each group in terms of expertise and disciplinary background, with up to 35 people participating in total
3. A brief email correspondence within each group to identify key issues and agree key reading for that group. This will include the present research protocol, a draft report of the rapid and systematic reviews and a report of the Delphi procedure

The purpose of the consultation meeting is for attendees to draft broad recommendations that will form the basis for guidance statements, in light of the background literature identified on measurement reactivity and the report of the Delphi procedure to inform the scope of the guidance. The groups will first work on key topics to produce broad draft recommendations; these will then be presented to the whole group for detailed plenary discussions. A record will be kept of the key gaps in the current evidence base. We will consider those gaps in existing evidence where it is not possible to develop guidance statements with a view to identifying priorities for future research.

After the meeting, a writing committee will consist of the MERIT study research team and meeting group leads. Each meeting group lead will be asked to produce text to describe the rationale for each guidance statement and provide elaboration and illustrative examples where helpful. This will be combined with agreed wording of the guidance statements and the general background sections prepared by the research team before the meeting, amended as appropriate. The draft guidance will be circulated to meeting attendees for at least one round of comments; ideally, all participants will be willing to endorse the guidance statements and the extent of endorsement will be checked for each guidance statement produced. A complete version of the guidance document will be agreed by the writing committee and sent to MRC/NIHR for further comment. This version will include appendices providing reports of the literature reviews and anonymised results of the Delphi procedure. The writing committee will respond to comments from the MRC/NIHR to produce a final version for publication.

## Discussion

The MERIT study aims to develop guidelines for how to minimise or avoid bias due to measurement reactivity in studies of interventions to improve health. With reference to relevant background scientific literature, MRC guidance will be developed in co-operation with experts in the field of health from many different scientific backgrounds. The face-to-face expert workshop will allow detailed content of the guidance to be developed in subgroups as well as group endorsement of each guidance statement produced. Guidance developed by several experts across many disciplines and institutions is more likely to be high impact, credible and become widely used.

Our ambition is to produce MRC guidance on measurement reactions in trials which will be used by future trial researchers, leading to the development of trials that are less likely to be at risk of bias. If there is insufficient evidence available to produce comprehensive guidelines, critical methodological research requirements will be identified. This work has significant policy implications for behaviour change interventions; many policy decisions on the roll out of population-level interventions rely on evidence from trials. It is important that this evidence reflects a range of perspectives. We expect the final version of the guidance to be published in early 2019.

## Additional file

Additional file 1: Word doc provided to show the categories of expertise sought for the MEasurement Reactions In Trials to improve health (MERIT) Delphi procedure. (DOCX 13 kb)

## Abbreviations

AMSTAR: Assessing the Methodological Quality of Systematic Reviews
CENTRAL: Cochrane Central Register of Controlled Trials.
CONSORT: Consolidated Standards of Reporting Trials
MERIT: Measurement Reactions in Trials
MRC: Medical Research Council
NIHR: National Institute for Health Research
PRISMA: Preferred Reporting Items for Systematic Reviews and Meta-Analyses
RCT: Randomised controlled trial
SMD: Standardised mean difference
UKCRC: UK Clinical Research Collaboration
