# Supplementary material for: Bias due to MEasurement Reactions In Trials to improve health (MERIT): protocol for research to develop MRC guidance
Source: Trials. 2018 Nov 26;19:653. doi: 10.1186/s13063-018-3017-5 (PMC6258480; doi:10.1186/s13063-018-3017-5)
Supplement: Supplementary file 1 — Word doc provided to show the categories of expertise sought for the MEasurement Reactions In Trials to improve health (MERIT) Delphi procedure. (DOCX 13 kb) [file 13063_2018_3017_MOESM1_ESM.docx]

**Bias due to MEasurement Reactions In Trials to improve health (MERIT): Protocol for research to develop MRC guidance.**

**Additional file 1**

*Categories of expertise sought for MERIT Delphi procedure*

Trial statistics

Trial conduct

Health Psychology/Behaviour Change

Epidemiology/Public Health

Sociology

Education/ Panel designs/ Ecological Momentary Assessment

Qualitative/mixed methods research

Measurement methodology

Measurement reactivity

Health economics

Evidence synthesis

Research Funding

eHealth

Lay/patient
